# Current teachers’ perceptions and students' perspectives regarding activities modalities, instructional settings during primary school physical education classes in China: a cross-sectional observational study

**DOI:** 10.3389/fspor.2024.1378317

**Published:** 2024-06-18

**Authors:** Jin Yan, Max Malkin, Jordan J. Smith, Philip Morgan, Narelle Eather

**Affiliations:** ^1^ School of Physical Education and Sports Science, Soochow University, Suzhou, China; ^2^ Centre for Active Living and Learning, University of Newcastle, Callaghan, NSW, Australia; ^3^ College of Human and Social Futures, University of Newcastle, Callaghan, NSW, Australia

**Keywords:** primary school, physical education, children, teaching, China

## Abstract

**Introduction:**

This study set out to understand the association between teaching practices, teacher confidence, competence, self-efficacy, and the resulting student outcomes.

**Methods:**

Data regarding teaching behaviours were collected via video recording and then evaluated using the MASTER Observation Tool. The information about demographics, self-reported teaching confidence, competence, self-efficacy, and student outcomes was collected using questionnaires. The association between teacher characteristics, and teacher and/or student outcome variables were tested using a one-way analysis of variance (ANOVA).

**Results:**

A total of ten primary schools were involved, including 597 children (age range: 10–12 years, grade 4–6) and 16 grade 4–6 PE teachers (with 16 PE classes). Most of the Physical Education (PE) lesson time was spent in training-form activities (60.2% ± 9.1), followed by instructional time (33.1% ± 8.6%), reflection (3.4% ± 2.3%), and warm-up (2.9% ± 2.0 %). It was observed that teaching behaviours and student outcomes were significantly better in urban than rural areas. Smaller class sizes (21–30 children) were found to have more positive feedback than larger ones (41–50 children). PE teachers with more than 10 years of teaching experience reported more teaching competence and self-efficacy than teachers with less than 10 years of experience. PE teachers with class sizes of 21–30 children enjoyed significantly better scores in self-efficacy than classes with 41–50 children. They also scored more highly in confidence and competence than classes with 41–50 and 51–60 pupils.

**Conclusion:**

The current study confirmed that teachers dedicated a large proportion of lesson time to PE delivering training-form activities, followed by instructional time. Teaching behaviour and student outcomes were associated with location and class size, but not gender. The study contributes to our understanding of PE instruction in Chinese primary schools and offers preliminary evidence to improve future PE teaching strategies in the country.

## Introduction

Physical education (PE) is a compulsory subject that offers unique opportunities for children to enhance their motor skills to actively engage in sports and physical activities (PA) during elementary (grades 1–6, students aged 5–12) and secondary school (grades 7–12, students aged 12–18) in many countries (1–5). Students' skill development is affected by curricular reasons, which would influence their performance during PE at school (6). Previous works examined student skill performance by conducting comprehensive and correct skill trials in PE classes (7, 8). Several studies have demonstrated that PE plays a vital role in the development of physical capability and movement-based competency (4, 9, 10). This enables children to participate in a wide range of PA and sports. Moreover, high-quality PE and sports programs can also contribute to students’ health and well-being and may significantly and positively impact their academic performance and engagement at school (11–15).

Schools are widely acknowledged as important for promoting PA and fitness in children and adolescents by offering opportunities to be active during and outside school hours (16, 17). However, numerous factors, including those related to the school institutions themselves but also teacher-related factors, have been shown to impact the quality of school PE programs adversely (18). For example, institutional barriers to delivering quality PE include insufficient equipment and facilities, a limited budget, negative attitudes toward PE among school executives, and inadequate professional development (19). Moreover, there are issues such as inadequate financial investment and funding support for PE, the lack of a knowledge base and high-quality teacher training programs, and decreasing school time devoted to PE (20, 21). In light of this, these issues would have a negative impact on both the effective and successful delivery of PE programs in schooling. Previous research has explored how PE instructors in schools approach instructional communication using a mixed methods approach, highlighting its significance as a fundamental aspect of teaching behaviour (22, 23).

PE is a compulsory course for all Chinese students from primary school to college (24, 25). In general, teachers with a degree in PE will be specifically recruited to teach the subject. In China, elementary school students take approximately four PE classes every week, with each class being 35–45 min long (26). The elementary PE course is aimed at developing students' athletic abilities and increasing their participation in sports. It is also important for mental and physical health, as well as social adaptability. In addition, the National Curriculum in PE offered PE teaching guidelines in 2011, including course goals and the learning evaluation system. Nonetheless, the PE activities and academic approaches used in each class are decided by teachers and schools according to the schools' actual circumstances (27). Furthermore, the Chinese government has been criticized for exclusively distributing the funds for *Ti Yu* (the umbrella term for anything sports-related) to elite sports competitions rather than to PE in schools (28).

In China, an administrative region with a population density greater than 1,500 people/km^2^ is considered an urban area, and its administration system is composed of town government, district government, and municipal government (29). Rural areas usually include villages and market towns, and the population is generally below 3,000 (30). In recent years, the gap between urban and rural China in terms of quality education and access to resources has become more pronounced. One of the most striking manifestations is the imbalance in PE. Tian et al. (31) found that when compared to rural areas, schools located in urban areas have significantly more funding, enabling them to build well-equipped sports facilities, employ specialist PE teachers, and provide a variety of extracurricular activities for students (including abundant opportunities for students to participate in physical activities and sport). Furthermore, Basch (32) highlighted that in rural areas where there are limited resources, schools are more likely to focus on academic subjects and pay PE teachers less, leading to the weakening of the status of PE. Consequently, urban students are more likely to explore sports opportunities outside school, such as joining clubs or signing up for private courses (32); and specialist PE teachers prefer to work in urban schools (33)—strengthening the divide between the delivery of PE in urban and rural areas in China (31).

Aligning with self-determination theory (SDT) and in the context of PE lessons, autonomous motivation can be enhanced when teachers provide students with choice, variety, and opportunities to contribute to lessons (34). According to SDT controlled motivation occupies an intermediate position on the motivation continuum and involves actions driven by rewards, fear of punishment, or avoidance of guilt (35). Controlled motivation and amotivation manifest when students engage in PE activities solely for rewards or due to external coercion. Previous research has demonstrated that controlled motivation and amotivation have been associated with negative outcomes for students, including reduced self-esteem, forgetting PE kits, and absenteeism from classes (36, 37).

Aligning with SDT, PE teachers can engage students in their lessons by inquiring about their interest in various sports and granting them the autonomy to select learning activities for specific lessons. Furthermore, teachers can provide guidance during students' participation in learning activities, offering a foundation to develop competence and overcome challenges. Encouraging teams to build and foster communication between teachers and students also facilitates a sense of belonging and connection among individual students. Similarly, the benefits of participating in quality PE include improved well-being, knowledge, performance, and persistence. This suggests that students respond well to their teachers' automatic support, with higher levels of stimulation and better PE achievements (35). In addition, previous studies have suggested that teachers' high self-confidence usually leads to diversified teaching strategies, which, in turn, helps students develop greater self-discipline and abilities, and the likelihood of successfully meeting learning outcomes in PE (38, 39).

Supporting the association between PE teacher characteristics and the physical education of Chinese primary school students, an observational study conducted by Zhou et al. (27) indicated that elementary students fell short of China's recommended 50% moderate and vigorous physical activity (MVPA) level, and teaching experience and teacher gender play important roles in MVPA time during PE. However, a range of other factors, such as PE lesson structure, class size, school location, and teacher and student self-perceptions, are commonly associated with and may influence PE quality. Therefore, the primary aim of this study was to observe and evaluate the teaching practices of teachers delivering PE in primary schools. The secondary aim of this study was to identify if there are any associations between teaching practices and behaviours, teachers' perceived confidence, competence, and self-efficacy, and student-level outcomes (i.e., enjoyment, motivation, well-being, and physical self-perception).

## Methods

### Participants and study design

This observational study was cross-sectional design and conducted in the Fall semester, between September and the end of December 2021 in Beijing, China. According to the specific geographical, demographic, and socio-economic levels of the districts (based on *China's Urban-Rural Integration Development Report: Beijing Volume* (40), 10 primary schools were randomly selected from 10 districts (including 5 urban and 5 rural areas) from Beijing, China. Study participants included 597 children (age range: 10–12 years) and one of their parents (either mother or father) provided valid information for all variables of this study. The study protocol and procedures were approved by the Human Research Ethics Committee of the University of Newcastle, Australia (H-2018-0006).

Participating schools were asked to recruit one or two PE teachers who were willing to facilitate the typical delivery of a normal PE class and be filmed during a specific period (with the filming date undisclosed to the participant). A total of 16 grade 4–6 PE teachers (with 16 PE classes) were willing to attend this study, which included varied physical activities (e.g., Basketball, Football, Volleyball, Gymnastics, Athletics, and Martial arts). Detailed information regarding the characteristics of participating schools is available in Table 1. All grade 4–6 students taught by their grade 4–6 teachers were eligible to participate in the study. Eligible participants were Grade 4–6 students (aged 10–12 years), without an illness or injury that would preclude them from fully participating in PE. Prior to enrolment, written informed consent was sought from the participating school principals, class teachers, and the students involved, as well as their parents/guardians. Only participants providing signed consent were eligible to participate in the study and complete the teacher and student.

**Table 1:** Characteristic of participated schools PE context and class content.

Place	Context	Content of class	Class size
School A (Urban), Beijing, China	Upper pupils (Grade 5, boys and girls aged 10–11), PE teacher A, Class took place in indoor basketball hall.	Basketball basic skills (e.g., dribbling and Disguised dribbling)	31 (Boys: 15, Girls: 16)
School B (Urban), Beijing, China	Upper pupils (Grade 6, boys and girls aged 11–12), PE teacher B, Class took place in the outside playground.	Gymnastics with a focus on skill training-roll forward	33 (Boys: 17, Girls: 16)
School C (Urban), Beijing, China	Upper pupils (Grade 6, boys and girls aged 12–13), PE teacher C, Class took place in the Indoor Stadium	Volleyball theme-based activities (e.g., digging and passing)	29 (Boys: 17, Girls: 12)
School D (Urban), Beijing, China	Upper pupils (Grade 5, boys and girls aged 10–11), PE teacher D, Class took place in the outside playground.	Athletics with a focus on throwing solid balls from head-to-head forward	44 (Boys: 20, Girls: 24)
School E (Urban), Beijing, China	Upper pupils (Grade 6, boys and girls aged 11–12), PE teacher E, Class took place in the outside playground.	Martial arts with a focus on the Horse stance and thrust punch, bow stance and thrust punch	33 (Boys: 17, Girls: 18)
School F (Urban), Beijing, China	Upper pupils (Grade 4, boys and girls aged 10–11), PE teacher E, Class took place in the outside playground.	Football theme-based activities with passing	27 (Boys: 14, Girls: 13)
School G (Rural), Beijing, China	Upper pupils (Grade 5, boys and girls aged 11–12), PE teacher G, Class took place in the outside playground.	Athletics with a focus on high jumping	34 (Boys: 15, Girls: 19)
School H (Rural), Beijing, China	Upper pupils (Grade 4, boys and girls aged 10–11), PE teacher H, Class took place in the outside playground.	Athletics with endurance running	40 (Boys: 12, Girls: 28)
School I (Rural), Beijing, China	Upper pupils (Grade 4, boys and girls aged 10–11), PE teacher I, Class took place in the inside playground.	Gymnastics with a focus on Low horizontal bar bending and overhanging	38 (Boys: 22, Girls: 16)
School J (Rural), Beijing, China	Upper pupils (Grade 5, boys and girls aged 11–12), PE teacher J, Class took place in the outside playground.	Basketball theme-based activities (e.g., Dribbling on the ground, dribbling on the move, passing, and shooting)	32 (Boys: 20, Girls: 12)

### Chinese PE setting

In elementary schools, PE classes are co-educational, with each lesson lasting for 35–40 min, and lessons are carried out in various indoor and outdoor areas. In general, a PE lesson consists of three parts: (a) warm-up and introduction under the guidance of the PE teacher; (b) teacher's instruction on related sports skills, and students' practice in sports skills; and (c) summary and students' cool-down.

### Procedure

The lesson observations were filmed using a camera (iPad 2021, USA) mounted on a tripod and attached to a wide-angle lens, and the camera was also connected to a wireless microphone system. The camera remained focused on the PE lesson for its entirety. PE teachers wore a lapel microphone and a hip-mounted radio transmitter (Sony UWP-D21, Japan) to enable verbal comments and video footage to be simultaneously recorded (41). Due to COVID restrictions, a volunteer teacher from the school was appointed as the videographer. After filming, the participating PE teachers completed a questionnaire online. Specifically, before participating in the survey, all the respondents were made aware of the purpose of the research project have known the research project. The results of giving assent or consent have been registered by a research assistant, and the results were included in the subject file and each file was identified with a numeric code; then, the information was recorded into a database, which is accessible to project staff with authorization. The data were collected from September to the end of December 2021. All the respondents were given clear instructions on how to fill out the questionnaire.

### Study measures

#### MASTER program

An education program known as MASTER, which focuses on a games-based curriculum, and is delivered in junior sporting contexts, has been successful in creating healthy outcomes in children (1, 42, 43). The foundation of the MASTER framework education program is “positive teaching/coaching”. The concept is fostered and promoted through game-based pedagogical practices; it targets six factors shown to promote health and well-being, enjoyment, motivation, and physical outcomes (cardiorespiratory fitness and cognitive function) in children. More information about MASTER can be found in the Supplementary Table S1. The MASTER education program was developed to address current gaps in PE teaching and sports coaching education (1, 42). Eather et al. (43) have demonstrated that youth sports coaches who make use of the MASTER framework, which is a user-friendly and evidence-based practical instrument for planning and delivering sports sessions, contribute significantly to players' enjoyment, well-being, motivation, and skills development. This framework has been tested in three formative studies in the delivery of sports—but it has not been tested in the delivery of a sport in a school setting.

The six core Principles of the MASTER Framework target instructional practices and behaviour shown to increase intrinsic motivation, engagement, and learning amongst learners through more enjoyable, active, and game-based sessions. Aligning with SDT, MASTER aims to increase intrinsic motivation for participation in PE/sport by targeting the three basic psychological needs of autonomy, relatedness, and competence. For example, the MASTER Framework encourages sports coaches/teachers to provide students with choice and input into learning activity design and delivery (Autonomy), provide learning activities that are sport-specific (relatedness) and game-based to replicate the skills and understanding required to succeed in a real game (competence).

The MASTER framework, though having been developed for use in sports settings, nonetheless might have significant value for evaluating the quality of teachers' instruction and student experiences in PE, as the outcomes the MASTER framework promotes within the sports domain are equally relevant to PE, for example, a recent pilot randomized controlled trial in Chinese PE primary school (1), MASTER education program has been successfully implemented to improve teachers and students' perceptions (e.g., teaching of confidence and competence, and motivation, enjoyment and wellbeing of PE).

#### Observational instrument

Table 2 outlines the observation measure used in this study. This observational study employed the MASTER Coach Observation Tool (a modified version of the Coach Analysis Intervention System (44), which has been successfully applied to football (pilot study) (42), netball (43), and football (Randomised Control Trial) (45) in Australia. This tool was used to evaluate teaching behaviours by employing notational analysis (Table 2). The objective was to assess changes in teaching with regard to practice state (% of activity time in training or playing form), and feedback (e.g., corrective, negative, positive, hustle, and punishment).

**Table 2:** Teacher behaviour assessment criteria.

Teaching practice	Description
Practice state (% of activity time)	
Playing form (PF)	Involves defence to produce decision making (Must involve active defence)
Training form (TF)	Involves a focus on movement pattern development through repetition (No defence or decision making)
Instructional time (IT)	Teacher providing instruction or explanation or feedback
Teaching Behaviours (Frequency of times)	
Positive (+)	Positive feedback from the teacher.
Negative (-)	Negative feedback from the teacher. e.g., sarcasm, frustration without reason, scolding.
Performance (P)	The teacher gives information on the movement pattern that caused the result.
Improvement (Imp)	The teacher gives information on how to adjust a movement pattern to cause a result.
Hustle (H)	Verbal statements or gestures linked to an effort to activate, quicken or intensify previously directed behaviour.
Questioned (Q)	The teacher questioned players to obtain an answer why action has occurred.

The first author and 10 trained research assistants (majoring in tertiary-level studies in physical education and sports pedagogy) used the MASTER Observation Tool to examine teaching behaviors in PE classes. The training was administered through workshops (one-week training, three times a week, totaling 10 h). To determine the initial reliability of the coding, the first author and a trained researcher independently co-coded 10% of the collected videos, which is based on the previously published MASTER studies (42, 43, 45). As a result, The inter-observer coding reliability for the MASTER categories was training form (92%), instructional time (92%), reflection (91%), and warm-up (91%), averaging 92%. Discussion regarding any discrepancies (e.g., two independent researchers disagreed on the quantities of PE teachers using positive feedback in one of the selected PE classes, with the first researcher arguing that the teacher used it 30 times in the class and the other independent researcher arguing that it was 39, after re-coding the video together, the two researchers finally reached an agreement (36 times)) was then conducted.

The MASTER checklist was used to evaluate the teachers' behaviours during the recorded PE lessons. The MASTER checklist was determined using an 18-item checklist, with items recorded on a 5-point scale (i.e., 1 = Not at all true to 5 = Very true). The use of academic lesson time, learning activity type, and teaching behaviours were recorded and coded (see Supplementary Table S2).

#### PE teacher feedback

The type and amount of feedback described as a percentage of general feedback provided by the teacher during evaluated lessons have been recorded. At the same time, the feedback has been categorized into descriptive performance, descriptive results, prescriptive to improve, negative, positive, hustle, and punishment. This analytical perspective used in this study was previously developed and tested in three formative and published MASTER studies (42, 45, 46).

#### PE teacher questionnaires

A targeted questionnaire was adapted from a questionnaire that had been applied in previous investigations involving PE delivery in schools (1, 43). During the questionnaire survey, the demographic information, such as gender, time length of teaching PE, and age, was collected and assessed as follows:

(a) Confidence to teach PE (via 27 items using a 5-point Likert scale ranging from 1= “not at all confident” to 5= “completely confident” e.g., My ability to explain game concepts relating to skilful movement and gameplay in PE……) (47, 48); In the current study, the Cronbach alpha was *α* = 0.88.
(b) Competence to teach PE (via 20 items using a 5-point Likert scale ranging from 1= “not at all confident” to 5= “completely competent” e.g., Lesson planning for PE…..) (49); In the current study, the Cronbach alpha was *α* = 0.86.
(c) Self-efficacy to teach (via 24 self-report items using a 5-point Likert scale ranging from ‘Not at all to ‘A great deal e.g., How much can you do to get through to the most difficult students?) (50). In the current study, the Cronbach alpha was *α* = 0.79.

#### Student questionnaires

All consenting students completed a questionnaire that collected the following information: Demographic information was gathered, including gender, age, ethnicity, parents' education level, the number of years attending PE, and location.

The students were instructed to fill out the 10–15 min questionnaire independently. All the questionnaires adopted in the study had been translated independently into simplified Chinese by two bilingual scholar translators. Then, the Chinese version was back-translated to guarantee the equivalence of content meaning and the quality of translation (51). Minor discrepancies had been reconciled by discussions. The final version of the surveys adopted was confirmed by the translators. Although a pilot test of the translated survey was not conducted, the reliability of this approach has been employed in previous study in the Chinese context (52).

(a) An adapted version of the Physical Activity Children's Enjoyment Scale (PACES) was used to evaluate Sports Enjoyment (through 16 questions, such as I feel happy during sports training) (53). Potential scores are 16–80, in which a higher score suggests a higher level of enjoyment. It has been verified to be applicable to kids in China (Cronbach's *α*=0.91) (54).
(b) The Warwick-Edinburgh Mental Well-being Scale (WEMWBS) was adopted to assess well-being. To be specific, it included 14 questions (such as I have felt optimistic about the future in the last 2 weeks) (55). Possible scores are 14–70, in which a higher score suggests a higher well-being degree. This scale and items have presented good convergent and divergent reliability in China's kids (Cronbach's *α* = 0.889) (56).
(c) The Behavioral Regulation in Sport Questionnaire (BRSQ) was used to evaluate the motivation (57, 58). Specifically, it included 23 items, with the use of a 5-point Likert Scale (1= “disagree significantly” to 5= “agree greatly” For example, I am in PE given that the benefits are crucial for me). It has been verified to be applicable to China's kids (*χ*2/df (3.2), CFI (.88), and RMSEA (.07)) (52).
(d) Physical self-perception was assessed using the athlete subscale of the Self-perception Profile for Children and consists of 6-questions (e.g., “Some kids do very well at all kinds of sports” (1), which has previously demonstrated validity and reliability in Chinese grade 4–6 children (*χ*2 = 1,084.18, df = 384, *χ*2/df = 2.82, RMSEA = 0.063, NFI = 0.84, NNFI = 0.88, CFI = 0.90, IFI = 0.90, GFI = 0.86) (59).

### Data analysis

Data were coded and quantified for each type of teacher behaviour and practice activity. Subsequently, the data were organized by using two methods: analysis of the entire physical education class and analysis of individual teacher feedback. Regarding the analysis of the entire physical education class, the frequency and duration of each coded category were calculated. Researchers studying teaching/coaching behaviour have utilized frequencies and percentages to describe behaviours in previous related studies (27, 60, 61). However, since frequency data could be varied based on class duration, we figured up the frequency per hour and the percentage relative to the duration of the teaching class for each observed behaviour and practice activity. These values were gained by dividing the total event time by the total duration of the teaching class and multiplying the result by 100. Regarding the analysis of individual teacher feedback, we recorded the type and quantity of feedback (e.g., negative, positive, hustle, and punishment) given by the teacher during evaluated lessons, measured as the frequency per hour (45).

Our study adopted a correlational design as the primary research method and data from individual questionnaires were filtered for missing or implausible values. Beyond that, the Shapiro–Wilk test was performed to examine the normality of the data (62). Moreover, the characteristics of the sample were reported using descriptive statistics (mean/standard deviation and proportion). The independent variables were teaching practices and behaviours (see Table 2, e.g., positive or negative feedback) in different activity types and lesson contexts. The dependent variables were the teachers' perceived confidence, competence, and self-efficacy to teach, and students' enjoyment, well-being, motivation, and physical self-perception. Information on PE teachers' gender, age, number of years teaching PE and location, and children participants' gender, age, ethnicity, parents' education level, the number of years attending PE and location, and size of the class was measured by a self-reported questionnaire. All analyses were conducted using the Statistical Package for the Social Sciences (Version 26.0; IBM Corp., Corp. Armonk, NY, USA), and the alpha level was set at *p* < 0.05. One-way analysis of variance (ANOVA) and Least Significant Difference (LSD) was conducted to identify where the significant differences occurred. Partial eta-squared (*η*2) was used as a measure of effect size, with values of 0.04, 0.25, and 0.64 used to represent small, medium, and large effect sizes, respectively (63).

## Results

After checking the normality of the data, it can be found that the data follows a normal distribution. Characteristics of the study sample are provided in Table 3. In total, 623 children participated, 26 of whom did not provide valid responses or missing data (597, 95.8% response rate; 45.9% female, mean age = 10.5 ± 1.7); along with 16 full-time PE teachers (9 males, 56%; 7 females, 44%; mean age years = 31.6 ± 5.0; mean years teaching experiencing = 9.0 ± 2.3). There were approximately 640 min, a total of 16 PE lessons video footage used in the analysis.

**Table 3:** Characteristics of participants in this study.

	*N*	%
Overall (student)	597	100
Gender		
Boy	323	54.1
Girl	274	45.9
Grade group		
Grade 4	240	40.2
Grade 5	256	42.9
Grade 6	101	16.9
Ethnicity		
Han	562	94.1
Minority	35	5.9
Geographical location		
Urban	324	54.2
Rural	273	45.8
Parent education		
Pre-primary	125	20.9
Primary school	173	28.9
Secondary school	189	31.6
College and above	110	18.6
Number of years attending PE		
1 year	42	7.0
2 years	101	16.9
3 years	119	19.9
4 years	129	21.6
5 years	137	22.9
6 years	69	11.7
Overall (PE teacher)	16	100
Gender		
Male	9	56
Female	7	44
Age		
Mean years (SD)	31.6 (5.0)	
Number of years teaching PE		
1–5 years	2	12.5
6–10 years	7	43.75
Over 10 years	7	43.75
Mean years (SD)	9.0 (2.3)	
Size of class		
0–30	6	37.5
31–40	3	18.7
41–50	5	31.2
Over 50	2	12.6
Mean number (SD)	37.2 (2.3)	

### Current Chinese PE lesson structure

Altogether, 2,264 PE-specific teaching practice activities (e.g., positive, negative feedback, and performance, see Table 2) were recorded and coded, over more than 640 min of teaching time. On average, a lesson lasted 40.2 (SD = 3.2) min. As demonstrated in Table 4, the largest proportion of the teaching time was allocated to training-form activities (60.2% ± 9.1%), which was followed by instructional time (33.1% ± 8.6%), reflection, and warm-up only accounted for 3.4% and 2.9%, respectively.

**Table 4:** The mean percentage of time allocated to the different practice components of a whole teaching session.

			Total lesson		
			40 mins		
			% of time		
	*N*	Mini	Max	Mean	SD
Warm-up	16	0.0	8.0%	2.9%	2.0%
Playing form (PF)	16	0.0	0.0	0.0	0.0
Training form (TF)	16	44.0%	75.0%	60.2%	9.1%
Instructional time (IT)	16	18.0%	49.0%	33.1%	8.6%
Reflection:	16	0.0	8.0%	3.4%	2.3%

### MASTER checklist grading

The MASTER Checklist Grading, based on observations of PE lessons by the research team, is presented in Table 5. “Maximize Player Activity” was rated the highest score (4.5 ± 0.5) by two independent observers, followed by “Strengths-Based” (2.5 ± 0.8), “Reflection & Feedback” (2.4 ± 0.7), and “Thinking Players” (1.8 ± 0.5). “Activate Learning” and “Engagement” received the lowest rating of 1.6 ± 0.5 and 1.5 ± 0.5 respectively (Table 5).

**Table 5:** MASTER checklist grading (information and recording sheet).

	Rater 1	Rater 2	Mean
Adherence to MASTER principles	40.8 (2.3)	40.1 (2.6)	40.4 (2.4)
Maximize player activity			
The teacher provides clear, concise, and focused instructions and demonstrations^a^	4.7 (0.4)	4.3 (0.4)	4.5 (0.4)
The teacher engages all players in session activities^a^	4.4 (0.5)	4.4 (0.4)	4.4 (0.5)
Activate Learning			
WHAT (is the game, or skill/s being learned and developed)^a^	1.4 (0.5)	1.5 (0.4)	1.5 (0.5)
WHY (should players play the game, or develop the skill/s)^a^	1.3 (0.4)	1.2 (0.6)	1.3 (0.5)
WHEN &/or WHERE (are the skills being developed used in the game)^a^	1.4 (0.4)	1.7 (0.4)	1.6 (0.4)
HOW (do you perform this well—what is a quality performance?)^a^	2.1 (0.7)	1.8 (0.7)	2.0 (0.7)
Strengths-based			
The teacher optimizes the challenge within training sessions for all players^a^	2.7 (1.0)	2.4 (1.2)	2.6 (1.1)
Teacher is positive^a^	3.5 (0.8)	3.4 (0.9)	3.5 (0.9)
The teacher promotes an “attempt is success” mindset^a^	1.4 (0.6)	1.7 (0.6)	1.6 (0.6)
Thinking players			
The teacher uses opposed activities (i.e., one or more defenders)^a^	1.3 (0.4)	1.4 (0.4)	1.4 (0.4)
The teacher uses questioning to facilitate learning^a^	2.0 (0.6)	1.7 (0.7)	1.9 (0.7)
Teacher promotes creativity^a^	2.2 (0.6)	2.0 (0.5)	2.1 (0.6)
Engagement			
The teacher has a ‘presence’ (e.g., voice projection, energy, humour)^a^	1.4 (0.5)	1.5 (0.4)	1.5 (0.5)
The teacher uses varied, challenging, relevant, and enjoyable activities^a^	1.7 (0.6)	1.6 (0.7)	1.7 (0.7)
The teacher utilises a ‘hook’ to engage players/students^a^	1.2 (0.5)	1.3 (0.4)	1.3 (0.5)
Reflection & feedback			
The teacher facilitates player reflection^a^	2.2 (0.6)	2.0 (0.8)	2.1 (0.7)
The teacher uses reflection from the previous session to inform current and/or future sessions^a^	2.8 (0.7)	2.5 (1.1)	2.7 (0.9)
The teacher provides useful feedback and information to guide/improve future performances^a^	2.4 (0.4)	2.5 (0.5)	2.5 (0.5)

All values reported are the mean (SD).

^a^ On a 5-point scale ranging from not at all true (1) to very true (5).

### Differences in teaching behaviour by school characteristics

**Figure 1:**
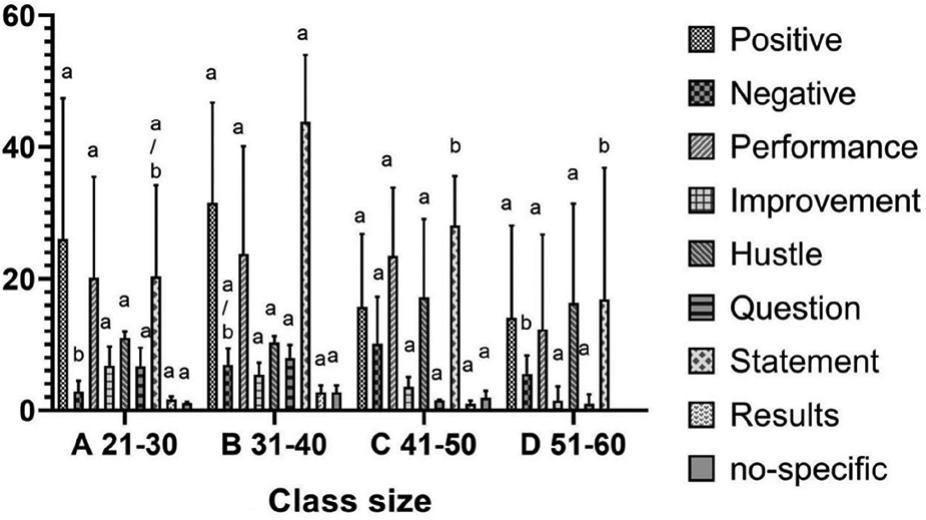
Teaching behaviour by different class size.

Table 6 displays the differences in the frequency of teaching behaviour by location and class size. Concerning location, the results of ANOVA revealed that the teaching behaviour of positive instructions [F_(1,14)_ = 20.266, *P* < 0.01, *η*2 = 0.59], negative instructions [F_(1,14)_ = 15.975, *P* < 0.01, *η*2 = 0.53], improvement instructions [F_(1,14)_ = 8.340, *P* < 0.05, *η*2 = 0.37], hustle instructions [F_(1,14)_ = 12.975, *P* < 0.01, *η*2 = 0.48] and question instructions [F_(1,14)_ = 24.054, *P* < 0.01, *η*2 = 0.63] in urban schools were significantly higher than in rural schools. In terms of class size, the ANOVA results revealed that negative teaching behaviour [F_(3,12)_ = 7.701, *P* < 0.01, *η*2 = 0.65] was associated with class size. The LSD tests indicated that class size with 21–30 children had significantly less frequency of negative instructions than class size with 41–50 children (Figure 1). However, no significant differences were observed in teacher behaviours according to teachers’ gender.

**Table 6:** Differences of teaching behaviour by location and class size in primary school PE classes.

	Location				Class size					
	Urban	Rural			21–30	31–40	41–50	Over 50		
	*M* (SD)	*M* (SD)	*F*	*η*2 Effect Size	*M* (SD)	*M* (SD)	*M* (SD)	*M* (SD)	*F*	*η*2 Effect Size
Positive	44.2 (11.5)	45.8 (4.8)	20.226**	0.59	41.1 (10.9)	42.3 (20.7)	23.6 (7.9)	24.0 (4.2)	2.907	0.42
Negative	4.6 (2.6)	22.4 (6.4)	15.975**	0.53	4.0 (1.6)	8.6 (5.1)	15.2 (5.0)	7.5 (3.5)	7.701**	0.65
Performance	33.7 (9.6)	13.1 (5.7)	1.148	0.07	31.0 (9.3)	35.3 (12.2)	30.8 (16.0)	22.5 (2.1)	0.448	0.10
Improvement	8.7 (4.1)	26.5 (13.6)	8.340*	0.37	8.8 (4.6)	6.6 (4.0)	4.6 (2.5)	3.0 (0)	1.801	0.20
Hustle	10.2 (7.3)	4.0 (2.2)	12.975**	0.48	11.6 (10.3)	11.0 (9.5)	25.6 (8.8)	27.0 (5.6)	3.148	0.44
Question	9.7 (4.4)	2.0 (1.0)	24.054**	0.63	8.6 (4.6)	9.3 (6.4)	1.6 (1.3)	2.0 (0)	4.052	0.50
Statement	39.8 (22.5)	29.1 (19.3)	0.834	0.05	30.1 (10.6)	51.0 (36.5)	33.4 (22.6)	31.0 (2.8)	0.719	0.15

* *P* < 0.05.

** *P* < 0.01.

### Differences in teacher-related variables by school and teacher characteristics

Table 7 outlines the differences in teacher-perceived confidence, competence, and self-efficacy by school location and teacher gender. With regard to location, the results of ANOVA showed the teacher perceived confidence [F_(1,14)_ = 33.199, *P* < 0.01, *η*2 = 0.73], competence [F_(1,14)_ = 15.138, *P* < 0.01, *η*2 = 0.73], and self-efficacy [F_(1,14)_ = 17.428, *P* < 0.01, *η*2 = 0.55] were significantly higher in urban schools than rural schools. However, there were no significant differences in teachers' perceived confidence, competence, and self-efficacy by gender.

**Table 7:** Difference in teacher perceived confidence, competence and self-efficacy by the locationa and teacher gender in primary school PE classes.

	Location				Teacher gender			
	Urban	Rural			Male	Female		
	*M* (SD)	*M* (SD)	*F*	*η*2 Effect Size	*M* (SD)	*M* (SD)	*F*	*η*2 Effect Size
Teachers’ self-efficacy	85.2 (16.8)	56.3 (10.2)	17.426**	0.55	76.2 (23.1)	63.8 (13.6)	1.557	0.10
Teachers’confidence	87.3 (14.6)	47.1 (13.3)	33.199**	0.73	70.7 (27.0)	62.7 (22.6)	0.400	0.02
Teachers’ competence	67.5 (10.3)	49.7 (7.7)	15.138**	0.52	62.4 (13.8)	52.7 (9.8)	1.977	0.12

* *P* < 0.05.

** *P* < 0.01.

### Differences in teacher-related variables by teaching experience and class size

**Figure 2:**
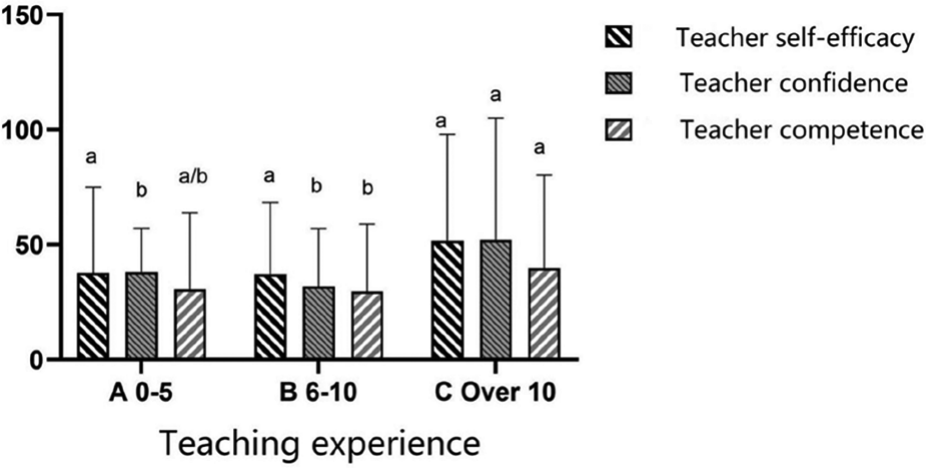
Teachers perceived self-efficacy, confidence and competence in teaching over the years.

Table 8 displays the differences in teacher-related variables according to years of teaching experience and class size. The results of ANOVA displayed that teacher-perceived self-efficacy [F_(2,13)_ = 4.151, *P* < 0.01, *η*2 = 0.39], confidence [F_(2,13)_ = 12.906, *P* < 0.01, *η*2 = 0.665], and competence [F_(2,13)_ = 6.186, *P* < 0.01, *η*2 = 0.48] made the significant teaching experience difference. The LSD tests illustrated that PE teachers with 1–5 years of teaching experience had significantly less confidence than teachers with over 10 years of teaching and teachers with 6–10 years of teaching experience had significantly less confidence than teachers with over 10 years of teaching. PE teachers with over 10 years of teaching experience had significantly more competence than the teachers with 6–10 years of teaching experience, and PE teachers with over 10 years of teaching experience had significantly more self-efficacy than teachers with 6–10 years of teaching experience (Figure 2).

**Table 8:** Differences of teacher perceived confidence, competence and self-efficacy by the teaching of years and class size in primary school PE classes.

	Teaching of years					Class size					
	1–5 years	6–10 years	Over 10 years			21–30	31–40	41–50	Over 50		
	*M* (SD)	*M* (SD)	*M* (SD)	*F*	*η*2 Effect Size	*M* (SD)	*M* (SD)	*M* (SD)	*M* (SD)	*F*	*η*2 Effect Size
Teachers’ self-efficacy	64.0 (11.3)	59.1 (15.3)	84.4 (18.8)	4.151**	0.390	87.6 (18.6)	68.3 (17.7)	55.2 (11.5)	63.0 (7.0)	4.153*	0.509
Teachers’ confidence	51.5 (24.7)	49.5 (14.0)	89.4 (14.6)	12.906**	0.665	89.8 (15.4)	65.0 (27.4)	47.8 (13.1)	51.5 (19.0)	5.827*	0.593
Teachers’ competence	54.0 (7.0)	50.2 (8.7)	68.2 (11.0)	6.186**	0.488	69.8 (10.9)	53.0 (13.2)	51.0 (4.9)	58.6 (12.7)	4.010*	0.501

* *P* < 0.05.

** *P* < 0.01.

**Figure 3:**
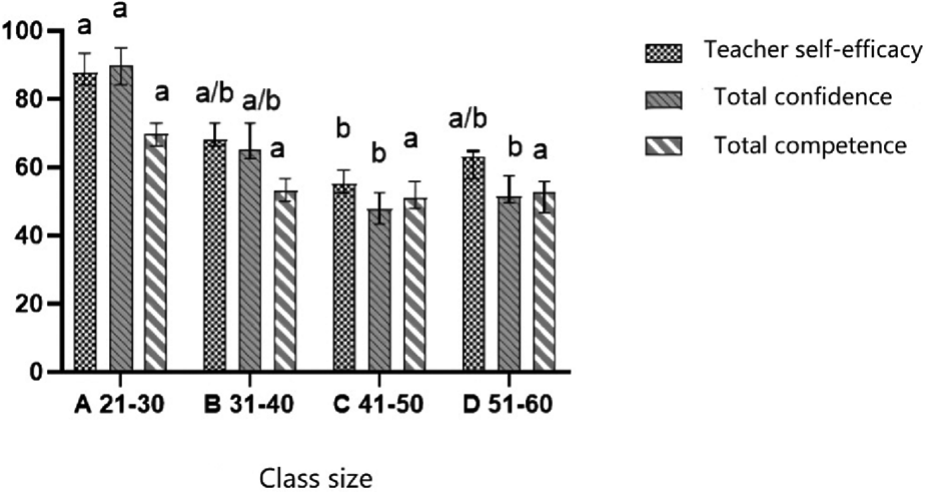
Teachers perceived self-efficacy, confidence and competence according to class size.

In terms of the class size, teachers perceived self-efficacy [F_(2,13)_ = 4.153, *P* < 0.05, *η*2 = 0.509], confidence [F_(2,13) _= 5.827, *P* < 0.05, *η*2 = 0.593], and competence [F_(2,13) _= 4.010, *P* < 0.05, *η*2 = 0.501] were significantly associated with varying class size. The LSD tests illustrated that class size with 21–30 PE teachers had significantly more scores in self-efficacy than class size with 41–50 children and more scores in confidence and competence than class sizes of 41–50 and 51–60 (Figure 3).

### Differences in student outcomes by school characteristics

**Figure 4:**
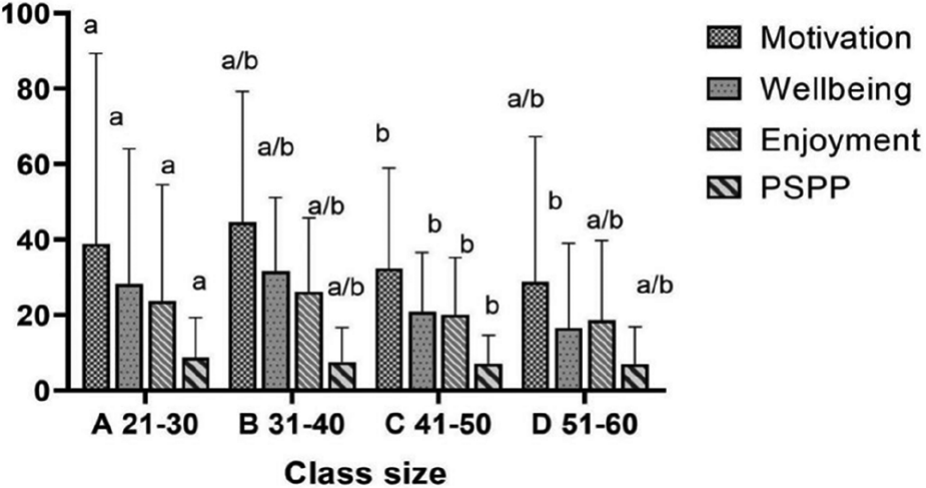
Student outcomes by location and class size.

Table 9 presents the differences in student outcomes by school location and class size. With regard to location, students' motivation [F_(1,14)_ = 36.288, *P* < 0.01, *η*2 = 0.72], well-being [F_(1,14)_ = 60.445, *P* < 0.01, *η*2 = 0.81], enjoyment [F_(1,14)_ = 29.735, *P* < 0.01, *η*2 = 0.68] and physical self-perception [F_(1,14)_ = 17.550, *P* < 0.01, *η*2 = 0.56] were significantly higher in urban compared with rural schools. In terms of class size, students' motivation [F_(3,12)_ = 4.272, *P* < 0.05, *η*2 = 0.51], well-being [F_(3,12)_ = 5.726, *P* < 0.05, *η*2 = 0.58], enjoyment [F_(3,12)_ = 3.824, *P* < 0.05, *η*2 = 0.48] and physical self-perception [F_(3,12)_ = 7.868, *P* < 0.05, *η*2 = 0.663] were significantly different according to class size. The LSD tests indicated that these outcomes were significantly greater in classes of 21–30 children compared with classes of 41–50 children (Figure 4). However, no significant differences were observed in student outcomes according to teachers' gender.

**Table 9:** Differences of student outcomes by location and class size in primary school PE classes.

	Location				Class size					
	Urban	Rural			21–30	31–40	41–50	Over 50		
	*M* (SD)	*M* (SD)	*F*	*η*2 Effect Size	*M* (SD)	*M* (SD)	*M* (SD)	*M* (SD)	*F*	*η*2 Effect Size
Motivation	76.0 (3.7)	51.7 (10.7)	36.288**	0.722	74.5 (3.0)	69.0 (19.9)	51.2 (13.5)	56.0 (1.4)	4.272*	0.516
Wellbeing	54.0 (2.6)	31.2 (7.8)	60.445**	0.812	53.5 (2.7)	45.3 (17.6)	32.0 (9.8)	32.5 (0.7)	5.726*	0.589
Enjoyment	45.8 (1.8)	30.8 (7.5)	29.735**	0.680	45.5 (1.8)	40.0 (12.1)	30.8 (9.3)	33.5 (3.5)	3.824*	0.489
PSPP	15.7 (1.2)	12.8 (1.4)	17.550**	0.556	16.1 (1.1)	14.0 (1.0)	12.4 (1.6)	14.0 (0)	7.868*	0.663

* *P* < 0.05.

** *P* < 0.01.

PSPP, physical self-perception.

## Discussion

The current study investigates the teaching practices of teachers delivering PE in Chinese primary schools and explores factors associated with teaching behaviors and student outcomes. We found that the predominant pedagogy of PE classes in Chinese primary schools was the delivery of training-form (skill-focused) activities and instructional time. Additionally, school location, class size, and teacher experience were significantly associated with teaching behaviours and student outcomes.

In response to our first aim, the majority of observed PE lesson time was spent in training-form activities (60.2%), followed by instructional time (33.1%). These results are in line with previous research, demonstrating that the majority of PE time spent in Chinese schools is devoted to the development of student motor abilities via repetitive practice, and PE teachers emphasize the role of training these skills in elementary schools (27, 64). Furthermore, our results show that children in China spend a large portion of PE lessons sedentary whilst PE teachers offer instruction, explanation, or feedback, as well as class administration and regulation. These findings are also supported by previous studies, whereby elementary school PE lesson time is largely spent on behavioral management and regulation (65, 66). Our findings demonstrate that teaching practices today have changed very little, and align with traditional delivery techniques used in early Chinese PE classes (in the 1904s) which were military-oriented (28). Skill-drill-type activities and quiet, obedient classes are expected within PE lessons in China (e.g., students talking to one another is forbidden, and they must stand in a line for most of PE class time (27). Therefore, teachers spend a significant amount of PE lesson time in practising skill techniques in highly structured and organized repetitive practice.

Furthermore, our findings show that school location plays an important role as a contextual variable impacting lesson delivery (67). Through our observation and assessment of PE lessons, urban PE provided more frequent positive comments and fewer negative hustle comments (e.g., sarcasm, frustration without reason, scolding) than teachers from rural areas. This result was consistent with earlier research (68), indicating that physical educators' judgments concerning curriculum and teaching are frequently different by school location. On the contrary, a study by Liu and Silverman (69) showed that, unlike physical educators in city areas, rural-based PE teachers were more likely to face obedient students who sat quietly, listened to the teacher, followed directions, and worked cooperatively with others. This may also explain why PE teachers' instructions in rural schools were less frequent than in urban areas in PE lessons. On the other hand, the present study also found that teachers perceived teaching confidence, competence, and self-efficacy in urban was significantly higher than in rural regions. This finding is supported by an earlier study, whereby teachers from urban schools in developed areas enjoyed a higher self-efficacy level compared with rural areas (70). Furthermore, economic factors may also contribute to the unequal investment in educational resources in urban and rural areas. For example, in China, less than two-fifths of the annual budget is allocated to the development of primary and secondary education; however, compared with rural primary schools, urban primary schools tend to receive most of the financial expenditure (71). In addition to PE development being affected by school location (urban vs. rural areas), community sports and club sports are also affected. Because the level of economic development and public funding in urban areas is generally higher, better sports infrastructure, such as orderly stadiums and well-maintained sports facilities, is tilted towards cities. These basic configurations not only represent opportunities for urban athletes to succeed but also attract more qualified coaches and trainers.

A positive association between class size and the frequency of negative feedback was found in this study. Although review-level evidence and experimental study indicated that class size might not have a significant impact on student's academic performance (72, 73). However, this finding contradicts Liu and Silverman (2006) (74) findings, who previously investigated the impact of different class sizes on teaching styles, primarily focusing on primary school students (aged 7–11). According to Blatchford's findings, students in smaller classes are more likely to receive individualized attention from teachers and an increased likelihood of receiving positive feedback (potentially raising the quality of the learning experience) (75). Similarly, it has been found by Chapman and Ludlow (76) that class size has a negative correlation with teachers using positive feedback during the classes, student performance, and learning interest. Potentially, as class sizes grow, a teacher's capacity to inspire each student to think and explore is diminished, because communication and interaction between teachers and students are reduced (77). More importantly, a past work conducted in Chinese background, implied that class size has a non-linear relationship with the improvement of student performance, and the best range of marginal benefits to measure the impact of class size on student performance is 21–30 students/class (78). This could be explained by the positive relationship between smaller class sizes and improving student confidence is mainly due to the increased personal attention of teachers and the increased feasibility of more personalized learning programs. As above mentioned, in small-class teaching, teachers have more opportunities to notice the changes or progress of students and are more likely to provide timely feedback based on finding out the strengths and weaknesses of students. This personalized approach not only shapes stronger teacher-student relationships but also creates an environment where students feel supported. Few systematic observations have been conducted to explore the difference between rural and urban students in PE. In one cross-sectional study involving 177 rural and 431 urban students, differences in location were associated with the basic psychological needs of children in PE classes (79). Specifically, Liu et al. (79) found that the autonomy, motivation, and enjoyment levels of urban students were significantly higher than that of rural students. It is possible that students in large cities have access to a wide range of sporting opportunities, whereas, in rural areas, PE is less appealing and restrictive (e.g., insufficient types of equipment to access sports). This may result in a lack of teacher-student interaction, which may lower students' interest in PE. Class size can also affect students' motivation, well-being, enjoyment, self-perception, and other factors during PE, in addition to geographical disparities. Prior research has identified that with the increase in class size, teachers' time for class management increased significantly (80). To illustrate, in primary school classes with less than 20 students, classroom management takes up 19.5% of classroom time, while in classes with 60 students, this figure rises sharply to 28.2% (81). This evidence supports the recommendation from the National Association for Sport and Physical Education (NASPE) in America to ensure the teacher-student ratio is below 25 students per teacher.

In terms of teaching experience, results from the current study indicated that PE teachers with over 10 years of teaching experience had significantly more positive self-perceptions (confidence, competence, and self-efficacy) than teachers with 1–5 years and 6–10 years of teaching. Our results are consistent with several studies that suggested teaching experience might boost instructors' expertise, confidence, and effectiveness in delivering PE courses and teachers (18, 82, 83). This can be interpreted as teaching experience may improve teachers' efficiency in managing PE lessons and thus teaching confidence. In addition, rich teaching experience can also help interpret students' learning, provide feedback based on student performance, guide students to acquire new information, and help them seek alternative solutions when they encounter obstacles, studies have shown that more experienced teachers are more effective, frequent use of a student-centered approach (84).

The MASTER Framework was designed by Eather, Jones, et al. (2020) (42), to help sports coaches and PE teachers create a positive learning environment and facilitate the effective delivery of sports and PE by using a games-based approach. Results from the MASTER Checklist assessment demonstrated that the MASTER principles that were most poorly taught were “Thinking Players”, and “Engagement”, which rated only 1.4 and 1.5 out of a possible 5 respectively. “Thinking Players” refers to the teacher involving children in cognitive challenges via questioning to facilitate learning and learning activities that promote decision-making and creativity. Engagement refers to the teacher having a “presence” (e.g., voice projection, energy, humour), and using varied, challenging, relevant, and enjoyable learning activities. Previous studies highlighted that establishing game-related exploratory learning activities or employing game-based approaches that cater to individual complexities and dynamic learning environments is recommended for facilitating high-quality learning in children and adults at varying levels of physical activity (85, 86). Given that Chinese primary school PE classes have, a large focus on explicit instruction of traditional linear activities (skill drills) via traditional pedagogical practice, the application of game-based approaches in Chinese schools may help to increase children's learning, motivation, engagement, and success in PE.

### Limitations and future research directions

Several limitations should be acknowledged. Due to the COVID-19 pandemic in many regions in China, the possibility that the present study may not be completely generalisable, and this study was only carried out in 10 districts in the Beijing area and might not be representative of other regions in China, such as some remote areas, low-income, and rural areas. As a result, additional large-scale observational studies should be conducted nationwide. Second, this study used self-reported measures to obtain data on all the instructors' assessed confidence, competence, and self-efficacy, as well as student outcomes, therefore, these measures are prone to recall bias and social desirability bias. Thirdly, our study only examined the mental health outcomes of students. The influence of other factors, such as movement behaviours and quality, physical activity levels, and intensity in PE lessons were not assessed, which should be involved in future studies. Finally, the current study is cross-sectional, rather than assessing any causality of relationships between teacher-related factors and student outcomes during PE lessons in China, and the single observations may or may not have been representative of what occurred typically, but logistical constraints precluded additional recording of lessons. Therefore, intervention studies may help to understand the nature of the relationship between variables in future studies, and future longitudinal data are required to confirm/refute the findings to inform school education.

## Conclusion and practical implications

Our study found that teachers dedicated a large proportion of lesson time to PE delivering training-form activities, followed by instructional time. Teaching behaviour and student outcomes were associated with location and class size, but not gender. The present study contributes to our understanding of the delivery of PE in Chinese primary schools and provides preliminary evidence to inform future PE interventions, policies, and practices.

Given these findings, our study recommends several possible suggestions for forming an effective professional development program for PE teachers and the responsibility of the government in addressing the gap between urban and rural quality sports in China. There is a need for effective game-based approach (GBA) professional learning for both pre-service and in-service teachers. The research results suggest that a significant portion of PE class time is devoted to training-form activities, indicating a prevalent preference for a skills-oriented approach among in-service teachers in China (despite the evidence to support the benefits of GBA in PE and sport delivery) (87). To enhance teachers' understanding of GBA, it is suggested that the professional learning programs incorporate a range of essential tactical knowledge, instruction on game creation and modification, and increased demonstrations of GBA teaching. Besides, Other educational goals, such as motor, cognitive, and social development of PE classes, should be measured/evaluated in future studies to determine how teaching behavior is related to these outcomes. Finally, the government should be able to increase the basic salary of rural physical education teachers and provide subsidies to remote and economically disadvantaged areas.

## Data Availability

The raw data supporting the conclusions of this article will be made available by the authors, without undue reservation.
