# Supplementary material for: Current teachers’ perceptions and students' perspectives regarding activities modalities, instructional settings during primary school physical education classes in China: a cross-sectional observational study
Source: Front Sports Act Living. 2024 Jun 18;6:1378317. doi: 10.3389/fspor.2024.1378317 (PMC11217334; doi:10.3389/fspor.2024.1378317)
Supplement: Supplementary file 1 [file Table1.docx]

**Table 1. MASTER framework and supporting evidence**

| MASTER  PE teachers use the MASTER framework to create a positive learning environment and deliver effective lessons using games-based approach strategies (Eather et al., 2020a, Eather et al., 2020b). | |
| --- | --- |
| Maximise player activity | 1. Coach provides clear, concise and focused instructions and demonstrations  2. Coach engages all players in session activities  Theory: By minimizing wait time during activities and student management time, coaches can allocate more time to players engaging in learning activities and developing a deeper understanding of important concepts, skills and ideas (Miller et al., 2016, Docheff et al., 2008). Selecting small-sided games or playing-form activities that maximise the involvement of all players have shown to optimise skill development, physical activity levels and physical activity intensity levels during training sessions (Hill-Haas et al., 2011, Miller et al., 2017).  Application: Minimise talk time (get moving quickly); provide instructions and demonstrations simultaneously; no long lines or waiting for a turn; minimise unnecessary coach interactions; evaluate and adapt activities quickly; use small-sided games. |
| Activate Learning | 1. WHAT (is the game, or skill/s being learned and developed)  2. WHY (should players play the game, or develop the skill/s)  3. WHEN &/or WHERE (are the skills being developed used in the game)  4 . HOW (do you perform this well – what is a quality performance?)  Theory: Through player-centred, inquiry-based approaches to coaching (i.e., game-based coaching), and a deep understanding of the game, coaches can develop game-play skills and abilities, increase player engagement and motivation, develop positive relationships between coach and players, and among players, and provide positive affective experiences of learning through sport (Light, 2004). In this approach, coaches should include dialogue/discussion, reflection and purposeful game play, and set high and explicit expectations to promote deep understanding of the game and facilitate active learning.  In this approach, coaches should include dialogue/discussion, reflection and purposeful gameplay, and set high and explicit expectations to promote a deep understanding of the game and facilitate active learning (Duda, 2013, Ryan and Deci, 2000).  Application: Identity what you want to achieve in your training session (e.g., strong accurate passes); select activities that specifically work towards your aims within a game context; have an understanding of why the skills are important for success in football and when it is used in in-game situations; use small-sided games that are adapted to suit your session aims (playing form activities); provide information & demonstrations on how to perform the skill correctly; use discussion and dialogue to promote reflection, sharing of ideas and creative strategizing. |
| Strengths-Based | 1. Coach optimizes the challenge within training sessions for all players  2. Coach is positive  3. Coach promotes “attempt is success” mindset  Theory: Highlighting what learners cannot do or requiring players to attempt/perform a skill in front of their team and coach can be very negative -particularly for junior players. However, taking a positive pedagogical approach and through game-based practice, mistakes provide opportunities for players to learn (rather than being used as controlling devices) (Light, 2012, Light and Harvey, 2017).  Using a positive view of mistakes in the learning process the coach facilitates both short and long-term player development, positive affective responses to participation, and the desire to remain in the sport (Smeeton et al., 2005, Vickers et al., 1999). Creating supportive sporting environments that promote social support for player learning and development also encourages all players (in an inclusive environment) to try hard in a climate of mutual respect (Det, 2006).  Application: Vary activities, rules or challenges within activities to accommodate all skill levels; provide positive and constructive feedback (i.e., Sandwich approach); use positive body language; set goal based on achievements, not mistakes/failures; promote success in a range of ways as a “win” not just scoring or winning a game; |
| Thinking Players | 1. Coach uses opposed activities (i.e., one or more defender)  2. Coach uses questioning to facilitate learning  3. Coach promotes creativity  Theory: Through games, coaches provide players with opportunities to explore, create, attempt/re-attempt, discuss and strategize with teammates and coach to solve game problems (Pill, 2014). Through games-based coaching, learning occurs when players engage with the (physical) learning environment (Dewey, 1933), participate in meaningful and complex problem solving and interact in the feedback cycle through substantial communication, dialogue/questioning and discussion (Curry and Light, 2007, Det, 2006, Light and Fawns, 2003).  Application: Have defenders in your activities (small-sided games); use activities that require players to make decisions; use questioning to facilitate and gauge understanding and learning; encourage creativity – new ways to achieve goals. |
| Engagement | 1. Coach has a “presence” (e.g., voice projection, energy, humour)  2. Coach uses varied, challenging, relevant and enjoyable activities  3. Coach utilizes a “hook” to engage players/students  Theory: Players are more likely to be engaged and motivated in sport training sessions, become self-regulated, sustain effort in training activities, and continue to play sport if they are having ‘fun’ and activities cater for what children want (fun, gameplay, success, skill development/mastery, play in a team, exercise) and what children need to maximise learning and reach their potential through sport (Balish et al., 2014, Côté and Gilbert, 2009, Newton et al., 2000). The coach has a role to play in the selection of training activities and their delivery to facilitate player enjoyment of sport and to make the sporting experience significant.  Application: Coach is confident, friendly, enthusiastic, relaxed (not stressed); coach interacts with players (asks questions, responds, gives advice/feedback/instruction); be organised and plan sessions that players want to join in (fun, provide learning/development opportunities, variety, choice, netball relevant); coach uses an age-appropriate approach/language. |
| Reflection | 1. Coach facilitates player reflection  2. Coach uses reflection from the previous session to inform current and/or future sessions  Theory: Reflection is an essential element of an authentic learning environment (Herrington et al., 2014), and developing the ability to think about why and what one does is considered vital for learning and development (Tabachnick and Zeichner, 1991) – this is important for player and coach development (Cushion et al., 2003, Gilbert and Trudel, 2001, Kovacs and Corrie, 2017).  Application: Coach uses questioning and discussion to reflect on activities/session aims/success with players; ask players to reflect on their successes and contributions; use the MASTER Framework to reflect on coaching practices/successes of the session; use the information gained to inform how you design and run future training sessions. |

**Table 2, Peer evaluation: MASTER Checklist (Information and recording sheet)**

| **Maximize player activity** | 1. **The teacher provides clear, concise, and focused instructions and demonstrations** | **1** | **2** | **3** | **4** | **5** |
| --- | --- | --- | --- | --- | --- | --- |
|  | 1. **The teacher engages all players in session activities** | **1** | **2** | **3** | **4** | **5** |
|  | **Application:** Minimise talk time (get moving quickly); provide instructions and demonstrations simultaneously; no long lines or waiting for a turn; minimize unnecessary Teacher interactions; evaluate and adapt activities quickly; use small-sided games. | | | | | |
|  | **Discussion points:** | | | | | |
| **Activate Learning** | 1. **WHAT (is the game, or skill/s being learned and developed)** | **1** | **2** | **3** | **4** | **5** |
|  | 1. **WHY (should players play the game, or develop the skill/s)** | **1** | **2** | **3** | **4** | **5** |
|  | 1. **WHEN &/or WHERE (are the skills being developed used in the game)** | **1** | **2** | **3** | **4** | **5** |
|  | 1. **HOW (do you perform this well – what is a quality performance?)** | **1** | **2** | **3** | **4** | **5** |
|  | **Application:** Identify what you want to achieve in your training session (e.g., strong accurate passes); select activities that specifically work towards your aims within a game context; have an understanding of why the skills are important for success in (your sport) and when it is used in-game situations; use small-sided games that are adapted to suit your session aims; provide information & demonstrations on how to perform the skill correctly | | | | | |
|  | **Discussion points:** | | | | | |
| **Strengths-Based** | 1. **The teacher optimizes the challenge within training sessions for all players** | **1** | **2** | **3** | **4** | **5** |
|  | 1. **Teacher is positive** | **1** | **2** | **3** | **4** | **5** |
|  | 1. **The teacher promotes an ‘attempt is success’ mindset** | **1** | **2** | **3** | **4** | **5** |
|  | **Application:** Vary activities, rules, or challenges within activities to accommodate all skill levels; provide positive and constructive feedback (i.e. Sandwich approach); use positive body language; set goal based on achievements, not mistakes/failures; promote success in a range of ways as a ‘win’ not just scoring or winning a game | | | | | |
|  | **Discussion points:** | | | | | |
| **Thinking Players** | 1. **Teacher uses opposed activities (i.e. one or more defender)** | **1** | **2** | **3** | **4** | **5** |
|  | 1. **The teacher uses questioning to facilitate learning** | **1** | **2** | **3** | **4** | **5** |
|  | 1. **Teacher promotes creativity** | **1** | **2** | **3** | **4** | **5** |
|  | **Application:** Have defenders in your activities (small-sided games); use activities that require players to make decisions; use questioning to facilitate and gauge understanding and learning; encourage creativity – new ways to achieve goals | | | | | |
|  | **Discussion points:** | | | | | |
| **Engagement** | 1. **The teacher has a ‘presence’ (e.g. voice projection, energy, humour)** | **1** | **2** | **3** | **4** | **5** |
|  | 1. **The teacher uses varied, challenging, relevant, and enjoyable activities** | **1** | **2** | **3** | **4** | **5** |
|  | 1. **Teacher utlises a ‘hook’ to engage players / students** | **1** | **2** | **3** | **4** | **5** |
|  | **Application:** Teacher is confident, friendly, enthusiastic, relaxed (not stressed); Teacher interacts with players (asks questions, response, gives advice/feedback/instruction); be organized and plan sessions that players want to join in (fun, provide learning/development opportunities, variety, choice, netball relevant); Teacher uses age-appropriate approach/language. | | | | | |
|  | **Discussion points:** | | | | | |
| **Reflection & Feedback** | 1. **Teacher facilitates player reflection** | **1** | **2** | **3** | **4** | **5** |
|  | 1. **Teacher uses reflection from previous session to inform current and / or future sessions** | **1** | **2** | **3** | **4** | **5** |
|  | 1. **Teacher provides useful feedback and information to guide / improve future performances** | **1** | **2** | **3** | **4** | **5** |
|  | **Application:** Teacher uses questioning and discussion to reflect on activities/session aims/success with players; ask players to reflect on their successes and contributions; use the MASTER Framework to reflect on Teaching practices/successes of the session; use the information gained to inform how you design and run future training sessions; provide players with information that will help them to improve future performances (i.e., relevant, positive and prescriptive – not negative or mistake focused). | | | | | |
|  | **Discussion points:** | | | | | |

BALISH, S. M., MCLAREN, C., RAINHAM, D. & BLANCHARD, C. 2014. Correlates of youth sport attrition: A review and future directions. *Psychology of Sport and Exercise,* 15**,** 429-439.

CÔTÉ, J. & GILBERT, W. 2009. An integrative definition of coaching effectiveness and expertise. *International journal of sports science & coaching,* 4**,** 307-323.

CURRY, C. & LIGHT, R. Addressing the NSW quality teaching framework in physical education: Is game sense the answer. Proceedings of the Asia Pacific Conference on Teaching Sport and Physical Education for Understanding, 2007. Citeseer.

CUSHION, C. J., ARMOUR, K. M. & JONES, R. L. 2003. Coach education and continuing professional development: Experience and learning to coach. *Quest,* 55**,** 215-230.

DET, N. S. W. 2006. Quality teaching in NSW public schools: A classroom practice guide (2nd ed.). *In:* DIRECTORATE., N. D. O. E. A. T. P. S. A. C. (ed.).

DEWEY, J. 1933. How We Think. A Restatement of the Relation of Reflective Thinking to the Educative Process, Boston etc.(DC Heath and Company) 1933.

DOCHEFF, D. M., WOODS, M. K. & ERWIN, H. 2008. Using good BEHAVIOR to improve the learning environment. *Journal of Physical Education, Recreation & Dance,* 79**,** 14-16.

DUDA, J. L. 2013. The conceptual and empirical foundations of Empowering Coaching™: Setting the stage for the PAPA project. *International Journal of Sport and Exercise Psychology,* 11**,** 311-318.

EATHER, N., JONES, B., MILLER, A. & MORGAN, P. J. 2020a. Evaluating the impact of a coach development intervention for improving coaching practices in junior football (soccer): the “MASTER” pilot study. *Journal of sports sciences,* 38**,** 1441-1453.

EATHER, N., MILLER, A., JONES, B. & MORGAN, P. J. 2020b. Evaluating the impact of a coach development intervention for improving coaching practices and player outcomes in netball: The MASTER coaching randomized control trial. *International Journal of Sports Science & Coaching***,** 1747954120976966.

GILBERT, W. D. & TRUDEL, P. 2001. Learning to coach through experience: Reflection in model youth sport coaches. *Journal of teaching in physical education,* 21**,** 16-34.

HERRINGTON, J., PARKER, J. & BOASE-JELINEK, D. 2014. Connected authentic learning: Reflection and intentional learning. *Australian Journal of Education,* 58**,** 23-35.

HILL-HAAS, S. V., DAWSON, B., IMPELLIZZERI, F. M. & COUTTS, A. J. 2011. Physiology of small-sided games training in football. *Sports medicine,* 41**,** 199-220.

KOVACS, L. & CORRIE, S. 2017. Building reflective capability to enhance coaching practice. *Coaching Psychologist,* 13**,** 4-12.

LIGHT, R. 2004. Coaches' experiences of Game Sense: opportunities and challenges. *Physical Education & Sport Pedagogy,* 9**,** 115-131.

LIGHT, R. & FAWNS, R. 2003. Knowing the game: Integrating speech and action in games teaching through TGfU. *Quest,* 55**,** 161-176.

LIGHT, R. L. 2012. *Game sense: Pedagogy for performance, participation and enjoyment*, Routledge.

LIGHT, R. L. & HARVEY, S. 2017. Positive pedagogy for sport coaching. *Sport, Education and Society,* 22**,** 271-287.

MILLER, A., CHRISTENSEN, E., EATHER, N., GRAY, S., SPROULE, J., KEAY, J. & LUBANS, D. 2016. Can physical education and physical activity outcomes be developed simultaneously using a game-centered approach? *European Physical Education Review,* 22**,** 113-133.

MILLER, A., HARVEY, S., MORLEY, D., NEMES, R., JANES, M. & EATHER, N. 2017. Exposing athletes to playing form activity: Outcomes of a randomised control trial among community netball teams using a game-centred approach. *Journal of Sports Sciences,* 35**,** 1846-1857.

NEWTON, M., DUDA, J. L. & YIN, Z. 2000. Examination of the psychometric properties of the Perceived Motivational Climate in Sport Questionnaire-2 in a sample of female athletes. *Journal of sports sciences,* 18**,** 275-290.

PILL, S. 2014. Informing game sense pedagogy with constraints led theory for coaching in Australian football. *Sports Coaching Review,* 3**,** 46-62.

RYAN, R. M. & DECI, E. L. 2000. Self-determination theory and the facilitation of intrinsic motivation, social development, and well-being. *American psychologist,* 55**,** 68.

SMEETON, N. J., WILLIAMS, A. M., HODGES, N. J. & WARD, P. 2005. The relative effectiveness of various instructional approaches in developing anticipation skill. *Journal of Experimental Psychology: Applied,* 11**,** 98.

TABACHNICK, B. R. & ZEICHNER, K. M. 1991. *Issues and practices in inquiry-oriented teacher education*, Citeseer.

VICKERS, J. N., LIVINGSTON, L. F., UMERIS-BOHNERT, S. & HOLDEN, D. 1999. Decision training: the effects of complex instruction, variable practice and reduced delayed feedback on the acquisition and transfer of a motor skill. *Journal of sports sciences,* 17**,** 357-367.
